# Variability in Estimated Glomerular Filtration Rate by Area under the Curve Predicts Renal Outcomes in Chronic Kidney Disease

**DOI:** 10.1155/2014/802037

**Published:** 2014-10-23

**Authors:** Szu-Chia Chen, Ming-Yen Lin, Teng-Hui Huang, Chi-Chih Hung, Yi-Wen Chiu, Jer-Ming Chang, Jer-Chia Tsai, Shang-Jyh Hwang, Hung-Chun Chen

**Affiliations:** ^1^Division of Nephrology, Department of Internal Medicine, Kaohsiung Medical University Hospital, Kaohsiung Medical University, 100 Shih-Chuan 1st Road, Kaohsiung 807, Taiwan; ^2^Department of Internal Medicine, Kaohsiung Municipal Hsiao-Kang Hospital, Kaohsiung Medical University, Kaohsiung 807, Taiwan; ^3^Faculty of Medicine, College of Medicine, Kaohsiung Medical University, Kaohsiung 807, Taiwan; ^4^Department of Public Health, Kaohsiung Medical University, Kaohsiung 807, Taiwan; ^5^Faculty of Renal Care, College of Medicine, Kaohsiung Medical University, Kaohsiung 807, Taiwan

## Abstract

Greater variability in renal function is associated with mortality in patients with chronic kidney disease (CKD). However, few studies have demonstrated the predictive value of renal function variability in relation to renal outcomes. This study investigates the predictive ability of different methods of determining estimated glomerular filtration rate (eGFR) variability for progression to renal replacement therapy (RRT) in CKD patients. This was a prospective observational study, which enrolled 1,862 CKD patients. The renal end point was defined as commencement of RRT. The variability in eGFR was measured by the area under the eGFR curve (AUC)%. A significant improvement in model prediction was based on the −2 log likelihood ratio statistic. During a median 28.7-month follow-up, there were 564 (30.3%) patients receiving RRT. In an adjusted Cox model, a smaller initial eGFR AUC%_12M (*P* < 0.001), a smaller peak eGFR AUC%_12M (*P* < 0.001), and a larger negative eGFR slope_12M (*P* < 0.001) were associated with a higher risk of renal end point. Two calculated formulas: initial eGFR AUC%_12M and eGFR slope_12M were the best predictors. Our results demonstrate that the greater eGFR variability by AUC% is associated with the higher risk of progression to RRT.

## 1. Introduction 

Chronic kidney disease (CKD) is an increasing worldwide public health problem associated with increased morbidity and mortality [1, 2]. Greater variability in renal function is associated with high mortality in CKD [3]. The mechanisms of variability in renal function are multifactorial, including intrinsic renal disease (e.g., renal microvascular disease or impaired autoregulatory mechanisms) and extrinsic factors (e.g., volume status and presence and severity of concurrent illness). However, only limited studies have demonstrated the predictive value of renal function variability in relation to renal outcomes in CKD patients.

Plotting slopes of 1/creatinine or estimated glomerular filtration rate (eGFR) versus time has been used as a tool for outcome variable in clinical studies [4]. eGFR slope has been identified as an independent risk factor for cardiovascular morbidity and mortality and progression to end-stage renal disease [5–10]. It is necessary to obtain the summarized information in the multivariate data by repeated measurement. Dynamic fluctuations in eGFR slope change may contribute to additional prognostic information beyond cross-sectional data. The area under the curve (AUC) computed with the trapezoidal formula is widely used as an approach to measure the dynamic and accumulating change of clinical laboratory parameters [11–14], but little is known about its application in the repeated measurements of eGFR variability. The eGFR AUC% formula by repeated measurements of eGFR was used as a measure of variability in renal function in our study. We hypothesize that eGFR AUC%, a method of estimating renal function variability, is a useful predictor for renal outcomes in CKD patients. Hence, the purposes of the present study are (1) to determine whether eGFR AUC% is associated with renal outcomes in progression to renal replacement therapy (RRT) and (2) to compare the predictive ability of renal outcomes using different methods of estimating renal function variability and eGFR slope in CKD stage 3–5 patients.

## 2. Subjects and Methods

### 2.1. Participants and Measurements

**Figure 1:**
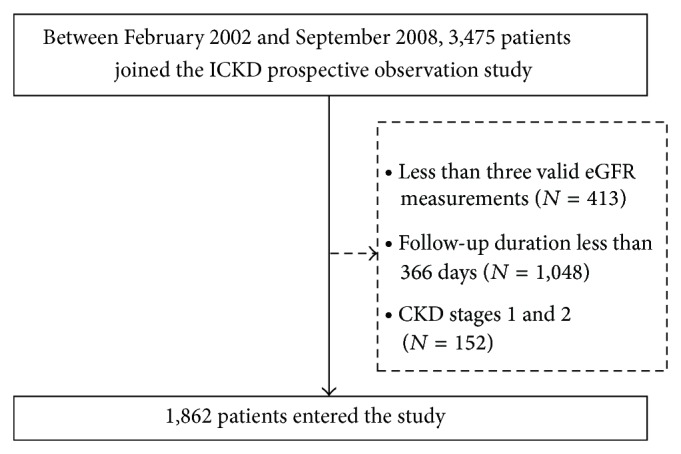
Derivation of patient cohort from all patients registered between February 2002 and September 2007; 1,862 patients were included in the analysis cohort.

Between February 2002 and September 2008, 3,475 patients who joined the ICKD (Integrated CKD care program) prospective observation study from two affiliated hospitals (Kaohsiung Medical University Hospital and Kaohsiung Municipal Hsiao-Kang Hospital) of Kaohsiung Medical University were included and followed until May 2010. CKD was defined by using the National Kidney Foundation-Kidney Disease Outcomes Quality Initiative (K/DOQI) guidelines, and the CKD stage was classified using each subject's baseline eGFR [15]. Patients who had only two serum creatinine measurements during follow-up (*n* = 413) or whose follow-up period was less than 12 months (*n* = 1048) were excluded. Besides, patients with CKD stages 1 and 2 (*n* = 152) were excluded. The final study population consisted of 1,862 CKD patients.  Figure 1 showed the flowchart of the derivation of the cohort. Baseline variables included demographic features (age and sex), medical history (diabetes mellitus [DM], hypertension, and cardiovascular disease), examination findings (body mass index [BMI] and blood pressure), and laboratory data (albumin, fasting glucose, triglyceride, total cholesterol, hemoglobin, total calcium, phosphate, calcium-phosphorous product [Ca × P product], uric acid, and urine protein-to-creatinine ratio). DM and hypertension were defined by clinical diagnosis. Cardiovascular disease was defined as clinical diagnosis of heart failure, acute or chronic ischemic heart disease, and cerebrovascular disease. The laboratory data 3 months before and after enrollment in the CKD care system were averaged and analyzed. In addition, information of medications using angiotensin converting enzyme inhibitors (ACEI) and angiotensin II receptor blockers (ARB) during the study period was obtained from medical records.

### 2.2. Quantification of Renal Function

Kidney function was quantified by using eGFR derived from the simplified Modification of Diet in Renal Disease (MDRD) Study equation. The equation was eGFR mL/min/1.73 m^2^ = 186 × Serum  creatinine^−1.154^ × Age^−0.203^ × 0.742 (if female) [16]. Serum creatinine was measured by the compensated Jaffé (kinetic alkaline picrate) method in a Roche/Integra 400 Analyzer (Roche Diagnostics, Mannheim, Germany) using a calibrator traceable to isotope-dilution mass spectrometry [17].

### 2.3. Definition of Renal End Point

The renal end point was defined as commencement of RRT. In patients reaching renal end point, renal function data were censored at the start of RRT. The other patients were followed until May 2010. The commencement of dialysis was determined according to the regulations by the National Health Service for dialysis therapy based on laboratory data, nutrition status, and uremic symptoms and signs.

### 2.4. Assessment of Rate of Renal Function Decline and Variability

**Figure 2:**
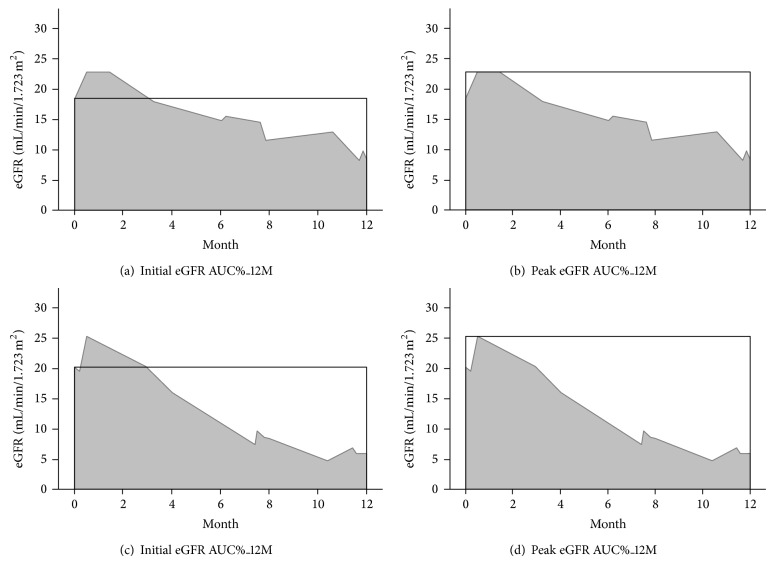
Two representative cases to illustrate the eGFR variability. In Case 1, the initial eGFR AUC%_12M (a) and peak eGFR AUC%_12M (b) were 85.0% and 68.9%, respectively. The eGFR slope of Case 1 was −1.06 mL/min/1.73 m^2^ per month. In Case 2, the initial eGFR AUC%_12M (c) and peak eGFR AUC%_12M (d) were 64.8% and 51.3%, respectively. The eGFR slope of Case 2 was −1.51 mL/min/1.73 m^2^ per month. Case 2 had greater eGFR variability than that of Case 1.

The rate of renal function decline was assessed by the slope of eGFR, defined as the regression coefficient between eGFR and time in units of mL/min/1.73 m^2^/year. At least three eGFR measurements were required to estimate eGFR slope. Faster renal function progression was reflected in a larger negative value of the slope. In addition, eGFR AUC% was used to estimate the variability in eGFR.  Table 1 showed the specifics of the calculations and illustrates two different methods of estimating eGFR AUC%. A smaller eGFR AUC% indicates greater eGFR variability. Initial eGFR AUC%_12M was defined as initial eGFR for baseline value and estimated variability in eGFR during 12 months. Peak eGFR AUC%_12M was defined as peak eGFR for baseline value and estimated variability in eGFR during 12 months.  Figure 2 represents two cases to illustrate the eGFR variability.

### 2.5. Statistical Analysis

Statistical analysis was performed using SPSS version 17.0 (SPSS Inc., Chicago, IL, USA) for Windows. Data are expressed as percentages, mean ± standard deviation, or median (25th–75th percentile) for triglyceride, urine protein-to-creatinine ratio, and days of follow-up. The number of all missing baseline data was less than 2% except the data of ACEI and/or ARB use. This adjustment of ACEI and/or ARB use was accomplished using multiple imputation approach. The differences between groups were checked by chi-square test for categorical variables, by independent *t*-test for continuous variables with approximately normal distribution, or by Mann-Whitney *U* test for continuous variables with skewed distribution. Cox proportional hazards analyses were used to investigate the relationships between different methods of estimating eGFR variability and eGFR slope with renal end point. The adjusted covariates included age, sex, a history of diabetes, hypertension, and cardiovascular disease, systolic and diastolic blood pressure, BMI, albumin, fasting glucose, triglyceride, total cholesterol, hemoglobin, eGFR, total calcium, phosphorous, CaXP product, uric acid, urine protein-to-creatinine ratio, ACEI and/or ARB use, and acute kidney injury episode. A significant improvement in model prediction was based on the −2 log likelihood ratio statistic, which followed a difference in likelihood ratio. The *P* value was based on the incremental value compared with the basic model. Differences were considered significant if the *P* value was less than 0.05.

## 3. Results

A total of 1,862 nondialyzed CKD patients were included. The mean age was 63.6 ± 13.4 years and there were 1,084 males and 778 females. The underlying etiologies of CKD in our patients included 646 with diabetic kidney disease (34.7%), 647 with nondiabetic glomerular diseases (34.7%), 200 cases of hypertension (10.7%), and 369 caused by other diseases (19.8%). The comparison of baseline characteristics between patients with and without renal end point was shown in Table 2. Compared with patients without renal end point, patients with renal end point were found to have a younger age, more female subjects, higher prevalence of DM and hypertension, higher systolic blood pressure, lower BMI, higher prevalence of advanced CKD stage, lower albumin, lower hemoglobin, lower baseline eGFR, lower calcium, higher phosphate, higher CaXP product, higher uric acid, higher urine protein-to-creatinine ratio, and higher prevalence of ACEI and/or ARB use. Besides, patients with renal end point had lower initial eGFR AUC%_12M, lower peak eGFR AUC%_12M, and lower eGFR slope_12M. The study patients were stratified into two groups according to median values of initial eGFR AUC%_12M (97.5%) and peak eGFR AUC%_12M (88.2%). The comparison of clinical characteristics among study groups was shown in Table 3.

### 3.1. Relation of eGFR Variability and eGFR Slope to Renal End Points

The mean follow-up period was 28.7 ± 14.0 months. During the period of follow-up, five hundred and sixty-four patients (30.3%) who started RRT were recorded in these 1,862 patients.  Table 4 showed the hazard ratios (HR) of different methods of estimating eGFR variability and eGFR slope for renal end point, with adjustments for age, sex, a history of diabetes, hypertension, and cardiovascular disease, systolic and diastolic blood pressure, BMI, albumin, fasting glucose, triglyceride, total cholesterol, hemoglobin, eGFR, total calcium, phosphorous, Ca × P product, uric acid, urine protein-to-creatinine ratio, ACEI and/or ARB use, and acute kidney injury episode. Patients with small initial eGFR AUC%_12M (HR, 0.962, 95% CI, 0.955 to 0.969; *P* < 0.001), small peak eGFR AUC%_12M (HR, 0.973, 95% CI, 0.966 to 0.980; *P* < 0.001), and negative eGFR slope_12M (HR, 0.358, 95% CI, 0.309 to 0.414; *P* < 0.001) were significantly associated with progression to renal end point. To clarify the effect of competitive risks between death and end-stage renal disease (ESRD), we further performed the multivariate analysis. We found that initial eGFR AUC%_12M (HR, 0.961, 95% CI, 0.954 to 0.968; *P* < 0.001), peak eGFR AUC%_12M (HR, 0.974, 95% CI, 0.967 to 0.981; *P* < 0.001), and eGFR slope_12M (HR, 0.279, 95% CI, 0.234 to 0.332; *P* < 0.001) were still significantly associated with progression to renal end point.

### 3.2. Incremental Values in eGFR Variability and eGFR Slope for Renal End Points

The incremental values in eGFR variability and eGFR slope, when they were added to the basic model to predict renal end point, were shown in Table 5. The addition of initial eGFR AUC%_12M (*P* < 0.001), peak eGFR AUC%_12M (*P* < 0.001), and eGFR slope_12M (*P* < 0.001) to the basic model significantly improved the predictive value of renal end points. The maximum change in the −2 log likelihood ratio was observed for initial eGFR AUC%_12M, followed by eGFR slope_12M, and peak eGFR AUC%_12M.

Besides, with regard to using standard error (SE) of the regression as a method of valuating eGFR variability, we found that the SE of the regression was not significantly associated with renal outcomes in multivariate analysis (HR, 0.926; 95% CI, 0.699 to 1.226; *P* = 0.590).

## 4. Discussion

In the present study, we evaluated the association between eGFR variability and progression to RRT and compared the predictive ability of renal outcomes using different methods to assess eGFR variability in CKD patients. We found that smaller eGFR AUC% or larger negative eGFR slope during 12 month follow-up periods was associated with a higher risk of progression to RRT and provided additional predictive value. Two calculated formulas initial eGFR AUC%_12M and eGFR slope_12M were the best predictors.

The first important finding of our study is the identification of greater eGFR variability as a risk factor for adverse renal outcomes in CKD. Previous studies have identified eGFR slope as an independent risk factor for cardiovascular morbidity and mortality [5–10]. Al-Aly et al. [3] evaluated the impact of variability in renal function on mortality in a large sample size of CKD patients (*n* = 51,304). They used the coefficient of variation of the regression line coefficient fitted to all outpatient measures of eGFR to define variability in renal function and found that patients in the highest tertile of eGFR variability had an increased risk of death, independent of both baseline level of eGFR and eGFR slope. In addition, such variability provided further prognostic information beyond baseline eGFR and eGFR slope [3]. The reason for higher mortality in patients with greater variability in eGFR might be related to factors both intrinsic and extrinsic to the kidney and unmeasured exposures that may threaten renal homeostasis (e.g., changes in intravascular volume status, congestive heart failure, liver or pulmonary disease, or medications). In addition, hospital-acquired and frequent episodes of acute kidney injury, diuretics use, and cardiorenal syndrome all contribute to greater eGFR variability. Our present study revealed that greater eGFR variability, expressed as smaller eGFR AUC%, was associated with progression to RRT and provided additional predictive value. This implies that variability in renal function is a potentially more important consideration in deciding which patients will progress to RRT.

The second important finding of our study is that one calculated formula initial eGFR AUC%_12M was a better predictor of adverse renal outcomes than eGFR slope. Unlike other summary measures (e.g., baseline, average, or maximum eGFR value), both pieces of information can be captured through the use of AUC [18]. The use of AUC simplifies the statistical analyses by transforming the multivariate data into a univariate variable, especially when many repeated measurement numbers exist and there is a need to summarize the information [14]. In addition, when the time interval between repeated measurements is not identical, the use of AUC provides an alternative approach to adjust for these differences [19]. O'Hare et al. investigated the variability in eGFR in the 2 years before initiation of dialysis in 5,606 Veterans Affairs patients and found that patients with greater eGFR variability were less likely to have received predialysis care and had a higher risk of death in the first year after dialysis [20]. An understanding of kidney function variability preceding dialysis can help guide clinical decision making (e.g., nephrology referral, vascular assessment, and transplant referral), goals of care, and anticipated service needs [20–22]. The formula for eGFR AUC% may also be used in long term follow-up in patients with CKD.

As for other approaches, such as using SE of the regression as a method of evaluating eGFR variability, our result is in agreement with Perkins RM's finding that eGFR variability by the SE did not predict ESRD outcome among CKD stage 3 patients [23]. Despite this finding, it needs further investigations to compare the significance of employing the different approaches for estimating eGFR variability.

In conclusion, our results demonstrate that the greater eGFR variability by AUC% is associated with the higher risk of progression to RRT. The formula of estimating eGFR variability by AUC method may provide a significant predictive value and guide the treatment strategies and choices in CKD patients.

## Figures and Tables

**Table 1:** Different methods to estimate variability of eGFR.

Method	Definition of calculation
(A) Initial eGFR AUC%_12M	Initial eGFR is baseline value and estimated variability of eGFR during 12 months (gray area was divided into rectangle area)

(B) Peak eGFR AUC%_12M	Peak eGFR is baseline value and estimated variability of eGFR during 12 months (gray area was divided into rectangle area)

eGFR: estimated glomerular filtration rate; AUC: area under the curve.

**Table 2:** Comparison of baseline characteristics between patients with and without renal end point.

Characteristics	Number of patients with data available	All patients (*n* = 1862)	Without renal end point (*n* = 1298)	With renal end point (*n* = 564)
Age (year)	1862	63.6 ± 13.4	64.4 ± 13.2	61.6 ± 13.7∗∗
Male gender (%)	1862	58.2	62.9	47.3∗∗
Diabetes mellitus (%)	1862	40.2	38.0	45.4∗
Hypertension (%)	1862	65.1	62.3	71.6∗∗
Cardiovascular disease (%)	1862	23.3	22.3	25.5
Systolic blood pressure (mmHg)	1862	138.0 ± 20.3	136.2 ± 19.9	142.1 ± 20.7∗∗
Diastolic blood pressure (mmHg)	1862	79.2 ± 12.4	79.0 ± 12.3	79.7 ± 12.8
Body mass index (kg/m^2^)	1861	24.9 ± 4.0	25.0 ± 3.9	24.6 ± 4.0∗
CKD stage	1862			
Stage 3 (%)		41.4	56.3	6.9∗∗
Stage 4 (%)		32.5	32.0	33.7
Stage 5 (%)		26.1	11.6	59.4
Laboratory parameters				
Albumin (g/dL)	1837	3.9 ± 0.5	4.0 ± 0.5	3.7 ± 0.5∗∗
Fasting glucose (mg/dL)	1842	116.4 ± 43.8	115.7 ± 42.7	118.2 ± 46.1
Triglyceride (mg/dL)	1847	124.5 (91–185)	123.5 (91–182.5)	127 (90.9–193)
Total cholesterol (mg/dL)	1846	196.5 ± 51.7	196.4 ± 51.7	196.6 ± 51.9
Hemoglobin (g/dL)	1858	11.3 ± 2.3	12.0 ± 2.2	9.8 ± 1.7∗∗
Baseline eGFR (mL/min/1.73 m^2^)	1862	27.2 ± 14.2	32.2 ± 13.1	15.6 ± 9.0∗∗
Total calcium (mg/dL)	1835	9.2 ± 0.7	9.3 ± 0.6	8.9 ± 0.9∗∗
Phosphorous (mg/dL)	1839	4.2 ± 1.0	3.9 ± 0.8	4.9 ± 1.1∗∗
CaXP product (mg^2^/dL^2^)	1832	38.4 ± 8.9	36.3 ± 7.4	43.4 ± 9.8∗∗
Uric acid (mg/dL)	1846	7.8 ± 2.0	7.7 ± 1.9	8.0 ± 2.0∗∗
Urine protein-to-creatinine ratio (mg/g)	1823	911.7 (362.3–1903.4)	613.2 (255.7–1290.6)	1871.2 (1047.1–3733.9)∗∗
ACEI and/or ARB use (%)	1757	45.9	44.5	49.2
Days of follow-up (days)	1862	873.5 (572–1282)	954.5 (600–1372.5)	724 (520–1086.8)∗∗
Variability of eGFR methods				
Initial eGFR AUC%_12M	1862	96.8 ± 20.5	100.3 ± 21.3	88.5 ± 15.9∗∗
Peak eGFR AUC%_12M	1862	85.8 ± 9.9	88.2 ± 8.6	80.4 ± 10.5∗∗
eGFR slope_12M	1862	−0.17 ± 0.03	−0.07 ± 0.05	−0.40 ± 0.03∗∗

CKD: chronic kidney disease; eGFR: estimated glomerular filtration rate; CaXP product: calcium-phosphorous product; ACEI: angiotensin converting enzyme inhibitor; ARB: angiotensin II receptor blocker.

^*^ *P* < 0.05, ^**^ *P* < 0.001 compared with patients without renal end point.

**Table 3:** Comparison of baseline characteristics between patients with eGFR AUC%_12M ≤ median and eGFR AUC%_12M > median.

Characteristics	Initial eGFR AUC%_12M ≤ median (95.7%) (*n* = 931)	Initial eGFR AUC%_12M > median (95.7%) (*n* = 931)	Peak eGFR AUC%_12M ≤ median (88.2%) (*n* = 931)	Peak eGFR AUC%_12M > median (88.2%) (*n* = 931)
Age (year)	63.3 ± 13.3	63.8 ± 13.5	63.0 ± 14.0	64.1 ± 12.8
Male gender (%)	54.4	62.0∗	52.6	63.7∗∗
Diabetes mellitus (%)	43.2	37.3∗	47.0	33.4∗∗
Hypertension (%)	68.0	62.3∗	70.5	59.8∗∗
Cardiovascular disease (%)	25.5	21.2∗	24.2	19.2∗∗
Systolic blood pressure (mmHg)	140.3 ± 20.5	135.7 ± 19.9∗∗	140.1 ± 21.8	135.9 ± 18.4∗∗
Diastolic blood pressure (mmHg)	79.5 ± 13.0	78.9 ± 11.9	79.0 ± 13.4	79.4 ± 11.5
Body mass index (kg/m^2^)	24.6 ± 4.0	25.2 ± 3.9∗	24.8 ± 4.0	25.0 ± 3.9
CKD stage				
Stage 3 (%)	30.0	52.7∗∗	27.4	55.3∗∗
Stage 4 (%)	36.3	28.8	35.8	29.3
Stage 5 (%)	33.7	18.5	36.8	15.4
Laboratory parameters				
Albumin (g/dL)	3.8 ± 0.5	4.0 ± 0.4∗∗	3.8 ± 0.5	4.0 ± 0.4∗∗
Fasting glucose (mg/dL)	118.5 ± 45.4	114.3 ± 42.0∗	120.1 ± 49.5	112.7 ± 30.8∗∗
Triglyceride (mg/dL)	125.3 (91–184.8)	123.5 (91–186)	127 (93–187)	123 (89–183)
Total cholesterol (mg/dL)	197.9 ± 51.7	195.0 ± 51.8	196.9 ± 57.5	196.1 ± 45.2
Hemoglobin (g/dL)	10.7 ± 2.1	11.9 ± 2.3∗∗	10.6 ± 2.1	12.1 ± 2.3∗∗
Baseline eGFR (mL/min/1.73 m^2^)	23.7 ± 13.5	30.7 ± 14.1∗∗	22.8 ± 13.1	31.6 ± 14.0∗∗
Total calcium (mg/dL)	9.0 ± 0.8	9.3 ± 0.6∗∗	9.0 ± 0.8	9.3 ± 0.6∗∗
Phosphorous (mg/dL)	4.4 ± 1.0	4.0 ± 0.9∗∗	4.5 ± 1.1	3.9 ± 0.8∗∗
CaXP product (mg^2^/dL^2^)	40.0 ± 9.3	36.9 ± 8.1∗∗	40.0 ± 9.8	36.4 ± 7.2∗∗
Uric acid (mg/dL)	7.7 ± 1.9	7.8 ± 2.1	8.0 ± 2.1	7.6 ± 1.8∗∗
Urine protein-to-creatinine ratio (mg/g)	1300.7 (615.7–2622.4)	592.6 (249.2–1347)∗∗	1324 (575.1–2675.3)	615.3 (249–1340.8)∗∗
ACEI and/or ARB use (%)	47.9	43.9	49.0	42.9∗
Days of follow-up (days)	747 (533–1162)	993 (630–1395)∗∗	730 (523–1174)	1008 (648–1398)∗∗

Abbreviations are the same as Table 1.

^*^ *P* < 0.05, ^**^ *P* < 0.001 compared with patients with eGFR AUC%_12M ≤ median.

**Table 4:** Risk association between eGFR variability and renal end points using Cox proportion hazard model analysis.

Method	HR (95% CI)	*P* value
Initial eGFR AUC%_12M	0.962 (0.955–0.969)	<0.001
Peak eGFR AUC%_12M	0.973 (0.966–0.980)	<0.001
eGFR slope_12M	0.358 (0.309–0.414)	<0.001

Values expressed as hazard ratio (HR) and 95% confidence interval (CI).

Covariates in the multivariate model included age, sex, a history of diabetes, hypertension, and cardiovascular disease, systolic and diastolic blood pressure, body mass index, albumin, fasting glucose, triglyceride, total cholesterol, hemoglobin, eGFR, total calcium, phosphorous, CaXP product, uric acid, urine protein-to-creatinine ratio, ACEI and/or ARB use, and acute kidney injury episode.

**Table 5:** Predictive values of eGFR variability in relation to renal end points.

Method	Δ − 2log⁡ likelihood ratio	*P* value
Initial eGFR AUC%_12M	141.782	<0.001
Peak eGFR AUC%_12M	42.258	<0.001
eGFR slope_12M	136.870	<0.001

*P* value was based on the incremental value compared with the previous model which was adjusted for age, sex, a history of diabetes, hypertension, and cardiovascular disease, systolic and diastolic blood pressure, body mass index, albumin, fasting glucose, triglyceride, total cholesterol, hemoglobin, eGFR, total calcium, phosphorous, CaXP product, uric acid, urine protein-to-creatinine ratio, ACEI and/or ARB use, and acute kidney injury episode.
